# Island Aging in the Ninth Decade and Beyond

**DOI:** 10.1093/geroni/igaa057.165

**Published:** 2020-12-16

**Authors:** Elaine Eliopoulos

**Affiliations:** PhD student, Weston, Massachusetts, United States

## Abstract

This novel research was designed to explore the lived experience of the aging body and its impact on social exclusion in remote island environments. Twenty three participants, aged 80 to 102 years old, in the Pacific Northwest, USA, were interviewed to explore the role their bodies played in everyday life. Despite presenting limited choices and lifestyle options, the island communities appeared to foster rather than impede a sense of competency and autonomy in the lives of these later life participants. Their ability to navigate their worlds, despite their physical limitations, was apparent in their reports of inclusion in the manner they desired. The island community context, characterized by low technology and mostly face to face encounters for goods and services, presented the participants with the possibility of accessible and meaningful engagement. Their sense of self and inclusion casts doubt on the dominant narrative of decline typically used to describe these late years. New narratives of corporeal being emerged from the data, demonstrating a complexity not captured by the singularity of the decline narrative. The visual methodology utilized enriched the depth and range of the semi-structured interviews, encouraging participants to think critically about their bodies. Possibilities for further research could explore the nuances of these new narratives and whether a different understanding of the older body may be useful.

